# Congenital talipes equinovarus: an epidemiological study in Sicily

**DOI:** 10.3109/17453674.2012.678797

**Published:** 2012-06-04

**Authors:** Vito Pavone, Sebastiano Bianca, Giuseppe Grosso, Piero Pavone, Antonio Mistretta, Maria Roberta Longo, Silvia Marino, Giuseppe Sessa

**Affiliations:** ^1^Orthopaedics Clinic, University of Catania; ^2^Genetics Unit, Garibaldi Hospital, Catania; ^3^“G.F. Ingrassia” Department – Section of Hygiene and Public Health, University of Catania; ^4^Pediatrics Unit, AOU OVE-Polyclinic, University of Catania, Italy.

## Abstract

**Background and purpose:**

Congenital talipes equinovarus (clubfoot) can present in 2 forms: “syndromic”, in which other malformations exist, and the more common “idiopathic” form, where there are no other associated malformations. We analyzed the epidemiology of congenital talipes equinovarus in the Sicilian population, looking for potential etiological factors.

**Patients and methods:**

Among the 801,324 live births recorded between January 1991 and December 2004, 827 cases were registered (560 males; M/F sex ratio: 2.1). Control infants were randomly selected from a historical cohort of live births without any major congenital malformations.

**Results:**

A positive family history of clubfoot, gender, and maternal smoking were found to be risk factors for clubfoot. Patients with clubfoot were born most frequently during the period January–March. No association was found between clubfoot and reproductive history, peri-conceptional maternal drug exposure, maternal education, or ethnicity.

**Interpretation:**

Our findings emphasize the importance of birth defects surveillance programs and their usefulness in investigating potential risk factors.

Congenital talipes equinovarus (CTEV), usually known as ‘clubfoot’, is the most common congenital orthopedic disease. Its etiology is unknown but appears to be complex (De Andrade et al. 1998, Chesney et al. 1999). The incidence is estimated to be 0.64–6.8 per 1,000 live births (Cartlidge 1984, Lochmiller et al. 1998, Wallander et al. 2006). However, in all epidemiological studies there are uncertainties created by variations in registration methods.

CTEV can present in 2 forms. In severe syndromic CTEV, other malformations are present such as spina bifida, spinal muscular atrophy, sacral agenesis, or arthrogryposis. Associated features may also include joint laxity, congenital dislocation of the hip, tibial torsion, ray anomalies of the foot, and the absence of some tarsal bones. In idiopathic CTEV—the more common form—the anomaly is isolated or may be associated with minor malformations. There may be a family history of foot anomalies.

The diagnosis of CTEV is based on clinical examination (irreducible equinus, varus of the hindfoot, adduction of the forefoot, cavus, and an “empty” heel pad) (Lochmiller et al. 1998, Wallander et al. 2006) and is straightforward. It can also be also diagnosed in utero by ultrasound (Danielson 1992, Sharma et al. 2011).

Studies on the epidemiology of CTEV could provide a basis for identifying potential risk factors. Thus, the aim of the present study was to determine the epidemiology of congenital talipes equinovarus in the Sicilian population and to describe the characteristics associated with the disease.

## Material and methods

### Data collection

Sicily, with a population of 5.6 million and about 50,000 births each year, had a Sicilian register of congenital malformations (ISMAC) between 1991 and 2004. In 2004, ISMAC was suspended and the register started again under new organization. We reviewed the database for notification of idiopathic CTEV. Case information included defect diagnosis, laterality of the defect, and presence of minor associated anomalies. In addition, data on infant and maternal health and demographics were collected. The infant variables collected included sex and birth weight, gestational age, and the number of months since birth. The maternal and pregnancy information collected included maternal and paternal age, family history, maternal history of pregnancy, exposure of the mother to illicit drugs or alcohol during the index pregnancy, maternal education, and ethnicity.

Control infants were randomly selected from a historic cohort of live births without any major congenital malformations; these infants had been enrolled previously to study other covariates in newborns (unpublished data). Controls were selected to achieve a ratio of 2 control infants per case, matched by year of birth.

### Statistics

To compare the annual number of newborns with congenital clubfoot with the total number of live births in Sicily between 1991 and 2004, we used official reports concerning native data from the Italian National Institute of Statistics (ISTAT). The cumulative incidence was calculated as the ratio between the number of children with clubfoot born each year and the number of native births during the same period. For the estimated incidence, the 95% confidence intervals (CIs) were thus calculated.

Differences in covariates of interest between the clubfoot group and the control group were tested using the Chi-square test for categorical variables and Student’s t-test or Mann-Whitney U-test for abnormally distributed continuous variables. Categorical data are presented as frequency and percentage while continuous data are reported as mean (SD).

To assess seasonal variation, we analyzed the monthly distribution of clubfoot deformities over the year (monthly cumulative incidence). The methodology of analyzing circular data has been described elsewhere (Zar 1999). In brief, we estimated one vector for children born with clubfoot and another vector for all other children born in Sicily during the same period. The average vector represents mean birth month for the children. The Rayleigh z-test (Fischer 1993) was used to test whether there was a uniform distribution of seasonal cumulative incidence for the children with clubfoot and for all other live births, respectively. To test whether the 2 distributions of month of birth (mean vector) were equal between the clubfoot group and other newborn children, the non-parametric Watson’s test was used (Zar 1999).

Unadjusted odds ratios (ORs) and the associated 95% CI for risk of clubfoot by different strata of maternal and paternal age, family history, pregnancy maternal history, maternal exposure to illicit drugs or alcohol during the index pregnancy, maternal education, and ethnicity were calculated. A multivariate logistic regression analysis was conducted to identify independent risk factors of clubfoot and adjusted ORs and associated 95% CI were calculated. A backward, stepwise selection approach using covariates found to be significant by univariate analysis was employed in the modeling strategy.

All statistical tests were two-tailed and a p-value of < 0.05 was considered significant. Variables found to be associated with the dependent variable in univariate analyses (probability threshold, p ≤ 0.10) were included in multivariate regression models. Data were entered into Microsoft Excel for Windows (Microsoft Corporation, Redmond, WA). Statistical analysis was performed using SPSS for Windows release 17.0.

## Results

Between January 1991 and December 2004, 827 cases (560 males, sex ratio 2.1) were recorded. Among the 801,324 newborns recorded in Sicily between 1991 and December 2004, the birth prevalence of children affected by clubfoot was 1.03 per 1,000 births (Table 1). The anomaly was bilateral in 529 cases (64%) and unilateral in 298 cases (36%)—55% of these on the right side. We also determined whether the distribution of patients with left or right side affected or bilateral clubfoot was similar in boys and girls. No sex-related side difference could be detected (p = 0.8) and the proportion of bilateral clubfoot was similar in boys and girls (64%) (p = 0.9) (Table 2).

**Table 1.** Number of children with idiopathic clubfoot in relation to total number of native births in Sicily between 1991 and 2004

	No. of children		Cumulative incidence
Year	Born alive	With clubfoot	per thousand (95% CI)
1991	62,189	70	1.12 (0.9–1.3)
1992	68,996	64	0.93 (0.8–1.2)
1993	64,873	73	1.12 (0.8–1.4)
1994	61,000	51	0.84 (0.6–1.2)
1995	58,063	55	0.95 (0.8–1.2)
1996	58,479	57	0.97 (0.8–1.2)
1997	57,629	70	1.20 (0.9–1.3)
1998	55,313	59	1.07 (1.0–1.3)
1999	54,879	58	1.05 (0.9–1.2)
2000	53,152	63	1.19 (1.0–1.3)
2001	51,890	51	0.98 (0.8–1.2)
2002	51,234	51	1.00 (0.8–1.2)
2003	51,899	53	1.02 (0.8–1.2)
2004	51,728	52	1.01 (0.8–1.3)
Total	801,324	827	1.03 (0.8–1.2)

**Table 2.** Distribution (%) according to sex, side, and bilateral occurrence of clubfoot

	Boys	Girls
Side	(n = 560)	(n = 267)
Unilateral	36	36
Left	45	46
Right	55	54
Bilateral	64	64

The seasonal variation in children with clubfoot peaked at 37º, corresponding to birth in the winter period (January to March) (Figure). The strength of this mean vector was, however, weak (r = 0.08).

**Figure:**
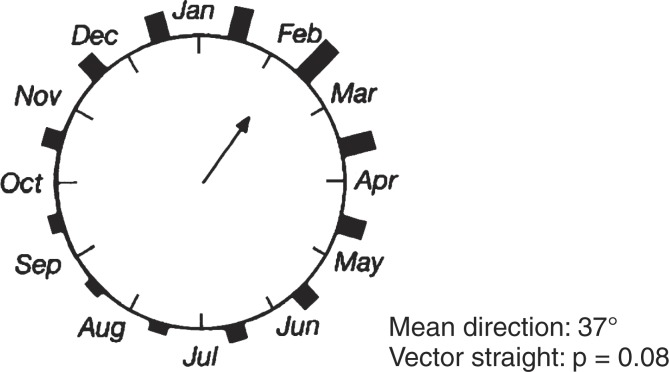
Circular histogram showing monthly distribution of new births with congenital clubfoot in Sicily during the period 1991–2004. The length of each black bar represents the frequency. The arrow indicates the mean vector corresponding to February.

When we compared the demographic characteristics (Table 3) of the children in the clubfoot group and of those in the control group, substantial differences were found for average mean birth weight (3,103 (SD 396) g in cases vs. 3,950 (360) g in controls), gestational age (39 (1.5) weeks vs. 39 (1.4) weeks in controls), maternal age (27 (4.9) years vs. 27 (3.7) years), and paternal age (32 (5.7) years vs. 31 (4.7) years). In 351 cases (43%), the child was the first-born and 0.5% of children had other associated minor abnormalities. A statistically significant difference was found for a positive family history of clubfoot, which was reported in 3.1% of patients (12 parents and 14 relatives) and in only 0.6% of the control group (p < 0.001).

**Table 3.** Descriptive characteristics of cases and controls, 1991–2004

	Cases	Controls	
Characteristic	(n = 827)	(n = 1,654)	p-value
Sex, %			0.001
Male	67.7	60.9	
Female	32.3	39.1	
Maternal race (%)			0.2
Caucasian	98	99	
Negroid	1	0.4	
Other	0.8	0.5	
Gestational age in weeks, mean (SD)	39 (1.5)	39 (1.4)	0.2
Birth weight (g), mean (SD)	3,103 (396)	3,950 (360)	0.6
Maternal age, mean (SD)	27 (4.9)	27 (3.7)	0.09
Paternal age, mean (SD)	32 (5.7)	31 (4.7)	0.1
Parity, %			0.6
1	42	41	
2	24	25	
3+	34	33	
Maternal smoking, %	26	16	< 0.001
Maternal drug exposure, %	52	51	0.5
Maternal education, %			0.3
≤ 8 years	48	50	
> 8 years	52	50	
Familiarity, %	3.1	0.6	< 0.001
Minor abnormalities **^a^**, %	0.5	0.2	0.3

**^a^** Minor abnormalities included hypospadia, clinodactily, low-set ears, angioma, few café au lait spots, transverse palmar crease, and white spot.

Univariate logistic regression revealed no association between clubfoot and reproductive history, peri-conceptional maternal drug exposure, maternal education, or ethnicity, but sex of the child, maternal smoking, and familial history of clubfoot were statistically significantly associated with higher risk of clubfoot (data not shown). After adjusting for covariates, sex (odds ratio (OR) = 0.68, CI: 0.56–0.81; p < 0.001), smoking (OR = 2.0, CI: 1.6–2.4; p < 0.001) and familial history (OR = 5.4, CI: 2.6–11; p < 0.001) were found to be independent predictors of clubfoot (Table 4).

**Table 4.** Crude and adjusted analysis of risk factors and 95% CI for clubfoot

	Univariate		Multivariate	
Characteristic	OR (95% CI)	p-value	OR (95% CI)	p-value
Gender				
Male	1		1	
Female	0.77 (0.62–0.89)	0.001	0.68 (0.56–0.81)	< 0.001
Maternal race				
Caucasian	1	0.2		
Negroid	2.3 (0.83–6.38)	0.1		
Other	1.6 (0.58–4.2)	0.4		
Gestational age	1.04 (0.98–1.1)	0.2		
Birth weight	1 (1–1)	0.6		
Maternal age	1 (0.99–1.04)	0.09		
Paternal age	1 (0.99–1.03)	0.1		
Parity				
1	1	0.6		
2	0.9 (0.72–1.11)	0.3		
3+	0.9 (0.82–1.2)	0.9		
Maternal smoking	1.8 (1.5–2.2)	< 0.001	2.0 (1.6–2.4)	< 0.001
Maternal drug exposure	1.1 (0.89–1.3)	0.5		
Maternal education				
≤ 8 years	1			
> 8 years	1.08 (0.92–1.3)	0.3		
Familiarity	5.3 (2.6–11)	< 0.001	5.4 (2.6–11)	< 0.001
Minor abnormalities	2 (0.5–8)	0.3		

## Discussion

We found a cumulative incidence of congenital clubfoot in Sicily during the period 1991–2004 that was similar to that reported for Italy (unpublished data). We also found a decrease in the cumulative incidence during the last years of the observation period. However, we cannot assess whether this is because of under-reporting. Although it is difficult to provide a definite answer, we consider our study to be important because it is one of the few studies to investigate cumulative incidence and prevalence of clubfoot with assessment of possible risk factors.

We found an incidence of CTEV of 1.03 per 1,000 live births, making it the fifth most common congenital malformation after cardiovascular anomalies (9 per 1,000), hypospadias (3.4 per 1,000), malformations of the urinary tract (2.4 per 1,000), and Down’s syndrome (1.5 per 1,000). CTEV was most often bilateral, and if unilateral, it more often involved the right side. Earlier studies have found the same (Gurnett et al. 2008, Dobbs et al. 2009, Parker et al. 2009). Moreover, we found that male sex was a strong risk factor, which also supports previous studies (Byron-Scott et al. 2005, Carey et al. 2005, Dickinson et al. 2008).

Seasonal variation in the incidence of idiopathic clubfoot has been reported, with different results (Barker and Macnicol 2002, Carney and Couburn 2005, Loder et al. 2006). The reasons put forward included temperature, food toxins, or seasonal viruses. We found a small increase in prevalence from January to March, as has already been reported (Pryor et al. 1991).

Although it has been widely accepted that clubfoot is multifactorial in origin, genetic factors clearly play a central role. In a study on identical twins, the concordance was 33% (Lochmiller et al. 1998). Moreover, nearly 25% of all cases are familial (Wynne Davies 1965). We also found that a family history of clubfoot was a risk factor. Additional evidence for a genetic etiology is provided by differences in the prevalence of clubfoot across ethnic populations, with a higher incidence of CTEV in Maoris than in New Zealanders of European origin (Sodre et al. 1990) and a higher incidence in Hungarian Gypsies than in non-Gypsies (Bellyei and Czeizel 1983). In Sicily, most immigrants are of African origin, but we found no effect of ethnicity.

Sharp et al. (2006) stressed that low folate status in pregnant women could cause several congenital malformations, and a reduction in the birth prevalence of idiopathic CTEV was observed in the United States and Denmark after fortification of grains with folic acid, or supplementation. Their study showed that subjects who carry the C677T variant of the methylene tetrahydrofolate reductase (MTHFR) gene, which has been implicated in the etiology of several congenital malformations including neural tube defects, have a reduced risk of idiopathic CTEV. This suggests that there is an interaction between maternal genotype and folate use during pregnancy. However, the assumption that folate has a protective role is still controversial: a study by Moorthi et al. (2005) conducted on a Texan population showed only a very small decrease in idiopathic CTEV after folic acid fortification of grains was introduced. Folate metabolism is complex, and many other nutrients and polymorphic genes could be involved in CTEV. Chapman et al. (2000) analyzed the role of a major gene and multifactorial inheritance in the etiology of CTEV in the Maori and Pacific Islander population by studying 287 affected families. Using the computer program POINTER, they showed that the best genetic model for CTEV in this population was a single dominant gene with a penetrance of 33% and a predicted gene frequency of 0.9%. Such results indicate the genetic predisposition to this anomaly, as confirmed by the results of the familial cases registered in our series.

Most previous studies have reported a correlation between maternal smoking during pregnancy and the incidence of CTEV, suggesting an important role for vascular disruption or compromise (Van den Eeden et al. 1990, Reefhuis et al. 1998, Dickinson et al. 2008, Parker et al. 2009). The role of maternal smoking could be linked to an abnormal arterial pattern in the affected foot, reduction in fetal and placental circulation due the vasoactivity of nicotine, and fetal tissue hypoxia induced by carbon monoxide (Honein et al. 2000). We found an adjusted odds ratio of 2 for any smoking during pregnancy, which is comparable with that previously reported.

Among other risk factors reported in the literature, early amniocentesis (in the thirteenth week of gestation) has been demonstrated to increase the risk of talipes equinovarus compared to chorionic villus sampling performed at the same gestational time (Farrell et al. 1999). Increased intrauterine compression was originally implicated as a risk factor but recent evidence does not appear to favor this hypothesis (Somppi 1984, Carney and Coburn 2005).

In Sicily, the number of children affected with clubfoot who are reported annually is decreasing despite an overall increase in congenital pathologies. It is likely there are multiple causes, with both genetic and environmental factors playing roles. At least in some cases, the phenotype may result from a threshold effect of different factors acting together. There is evidence that the development of bone, joints, connective tissue, innervation, vasculature, and muscle may each be implicated in the pathophysiology, and a disturbance of the overall process of medial rotation of the fetal foot may be the common pathway.

Our study should be considered in light of some limitations. First, there is a risk of underestimation of the true incidence due to missed cases. Our data were collected prospectively, however, and since practically all children in Italy are born in hospital, we believe that the number of cases missed was very small. Secondly, several important covariates (for example, birth characteristics such as early amniocentesis) were not included in our data.

To sum up, our findings emphasize the importance of birth defects surveillance programs and their usefulness in investigating potential risk factors. On the other hand, there is a lack of multiple surveillance programs in the Sicilian setting, as well as in other Italian regions. Given a national representative sample, multiregional studies are needed to provide greater statistical power to investigate risk factors and provide the opportunity to identify variation between different surveillance areas.
